# Multisensory Interactive Technologies for Primary Education: From Science to Technology

**DOI:** 10.3389/fpsyg.2019.01076

**Published:** 2019-06-28

**Authors:** Gualtiero Volpe, Monica Gori

**Affiliations:** ^1^ Casa Paganini–InfoMus, DIBRIS, University of Genoa, Genoa, Italy; ^2^ U-Vip Unit, Istituto Italiano di Tecnologia, Genoa, Italy

**Keywords:** multisensory technologies, education, integration of sensory modalities, enaction, inclusion

## Abstract

While technology is increasingly used in the classroom, we observe at the same time that making teachers and students accept it is more difficult than expected. In this work, we focus on multisensory technologies and we argue that the intersection between current challenges in pedagogical practices and recent scientific evidence opens novel opportunities for these technologies to bring a significant benefit to the learning process. In our view, multisensory technologies are ideal for effectively supporting an embodied and enactive pedagogical approach exploiting the best-suited sensory modality to teach a concept at school. This represents a great opportunity for designing technologies, which are both grounded on robust scientific evidence and tailored to the actual needs of teachers and students. Based on our experience in technology-enhanced learning projects, we propose six golden rules we deem important for catching this opportunity and fully exploiting it.

## Introduction

Multisensory education is conceived as an instructional method using visual, auditory, kinesthetic, and tactile ways to educate students (Joshi et al., 2002). There has been a longstanding interest in how learning can be supported by representations engaging multiple modalities. For example, the Montessori education tradition makes use of artifacts such as sandpaper letters children trace with their fingers to develop the physical skill of learning to write. Papert (1980) discussed the idea of body-syntonic learning – projecting an experiential understanding of how bodies move – into learning about geometry. Moreno and Mayer (1999) explored the cognitive impact of multimodal learning material in reducing cognitive load by representing information in more than one modality.

Technology entered the classroom many years ago. It can be considered as a medium for inquiry, communication, construction, and expression (Bruce and Levin, 1997). Early technological interventions consisted of endowing classrooms with devices such as overhead projectors, cassette players, and simple calculators. These devices were intended to support the traditional learning and teaching paradigms and usually did not enable direct interaction of students with technology. More recently, a broad palette of technological tools became available, including technologies for computer-assisted instruction, i.e., the use of computers for tutorials or simulation activities offered in substitution or as a supplement to teacher-directed instruction (Hicks and Holden, 2007), and for computer-based instruction, i.e., the use of computers in the delivery of instruction (Kulik, 1983). These technologies exploit devices, such as interactive whiteboards, laptops, smartphones, and tablets, which are mainly conceived to convey visual information and are not intended for embodied interaction. Walling (2014) argued that tablet computers are toolboxes for learner engagement and suggested that the transition to using tablet computers in education is a natural process for teenagers. For example, there are multiple applications for tablets for learning mathematics^1^. Falloon (2013) reviewed 45 apps selected by an experienced teacher. Of them, 27 were considered educational apps, which focused on a broad variety of topics, including numeracy skills, reinforcing spelling, acquiring new vocabulary, and improving phonetics. Nevertheless, these solutions usually rely on the ability to see digital content rather than physically interacting with it. At the same time, novel technological developments also enabled the use of multiple sensory channels, including the visual, auditory, and tactile ones. This technology has been defined as *multisensory technology*. Technological advances and increased availability of affordable devices (e.g., Kinect, Oculus Rift, and HTC Vive) allowed a fast adoption of multisensory technology in many areas (e.g., entertainment, games and exergames, and assistive technologies). Its introduction in the classroom, however, is still somewhat limited. Early works addressed the use of virtual reality in educational software for either enabling full immersion in virtual environments or accentuating specific sensory information (Raskind et al., 2005). Nowadays, technologies such as augmented reality (e.g., see Santos et al., 2014) and serious games (e.g., see Connolly et al., 2012) play a relevant role in many educational contexts, both in science and in the humanities. Multisensory technologies enabling embodied interaction were used, for example, to support teaching in computer programming (e.g., Katai and Toth, 2010; Katai, 2011), music (e.g., Varni et al., 2013), and dance (e.g., Rizzo et al., 2018). Baud-Bovy and Balzarotti (2017) reviewed recent research on force-feedback devices in educational settings, with a particular focus on primary school teaching. Less traditional tools were also exploited, including Job Access With Speech (JAWS) and Submersible Audible Light Sensor (SALS). JAWS is a computer screen reader program allowing blind and visually impaired users to read the screen. SALS is a glass wand with an embedded light sensor, enabling the measuring of color intensity changes. These tools were used in a science camp for visually impaired students (Supalo et al., 2011), but they could also be modified and adapted for general inclusion in multisensory education. Despite such initiatives and the growing interest in these tools, most often the introduction of multisensory technologies in the learning environment has been exploratory, piecemeal, or *ad hoc*, focusing on understanding the potentials of the different modalities, rather than taking a combined multisensory focus.

Stakeholders consider the adoption of a technology-mediated pedagogical approach as a must, and the quest for innovation heavily drives choices. Attempts at integrating technology in the classroom, however, do not often take into account the pedagogical needs and paradigms. Teachers and students are not involved in the innovation process, and development of technologies does not follow a proper evidence-based iterative design approach. The risk is that technology can be rejected. For example, Groff and Mouza (2008) presented a literature review on the challenges associated with the effective integration of technology in the classroom. More recently, Johnson et al. (2016) discussed common challenges educators face when attempting to introduce technology at school. Philip (2017) described the difficulties that were experienced in a project relying on novel mobile technologies in the classroom.

In this article, we argue that the intersection between current challenges in pedagogical practices and recent scientific evidence opens novel opportunities for acceptance of technology as a tool for education, and those multisensory technologies can specifically bring a significant benefit to the teaching and learning process.

## Scientific Evidence

The combination and the integration of multiple unimodal units are crucial to optimize our everyday interaction with the environment (Ernst and Bulthoff, 2004). Sensory combination allows us to maximize information delivered by different sensory modalities without these modalities being necessarily fused, while sensory integration enables reducing the variance in the sensory estimate to increase its reliability (Ernst and Bulthoff, 2004). In particular, sensory combination occurs when different environmental properties of the same object are estimated by means of different sensory modalities. Contrarily, sensory integration occurs when the same environmental property is estimated by different sensory modalities (Ernst and Bulthoff, 2004). Many recent studies show that our brain is able to integrate unisensory signals in a statistically optimal fashion as predicted by a Bayesian model, weighting each sense according to its reliability (Clarke and Yuille, 1990; Ghahramani et al., 1997; Ernst and Banks, 2002; Alais and Burr, 2004; Landy et al., 2011). This model has been useful to predict the multisensory integration behavior of adults across different sensory modalities in an optimal or near-optimal fashion (Ernst and Banks, 2002; Alais and Burr, 2004; Landy et al., 2011). There is also firm neurophysiological evidence for multisensory integration. Studies in cats have demonstrated that the midbrain structure superior colliculus (SC) is involved in integrating information between modalities and in initiating and controlling localization and orientation of motor responses (Stein and Meredith, 1993). This structure is highly sensitive to input from the association cortex, and emergence of multisensory integration critically depends on cross-modal experiences that alter the underlying neural circuit (Stein et al., 2014). Moreover, cortical deactivation impairs integration of multisensory signals (Jiang et al., 2002, 2007; Rowland et al., 2014). Studies in monkeys explored multisensory decision making and underlying neurophysiology by considering visual and vestibular integration (Gu et al., 2008). Similar effects were also observed in rodents (Raposo et al., 2012, 2014; Sheppard et al., 2013).

The role of sensory modalities in child development has been the subject of relevant research in developmental psychology, psychophysics, and neuroscience. On the one side, scientific results show that young infants seem to be able to match sensory information and benefit from the presence of congruent sensory signals (Lewkowicz, 1988, 1996; Bahrick and Lickliter, 2000, 2004; Bahrick et al., 2002; Neil et al., 2006). There is also evidence for cross-modal facilitation, where stimuli in one modality increase the responsiveness to stimuli in other modalities (Lewkowicz and Lickliter, 1994; Lickliter et al., 1996; Morrongiello et al., 1998). On the other side, the ability to integrate unisensory signals in a statistically optimal fashion develops quite late, after 8–10 years of age (Gori et al., 2008, 2012; Nardini et al., 2008; Petrini et al., 2014; Dekker et al., 2015; Adams, 2016). Recent results show that during the first years of life, sensory modalities interact and communicate with each other and the absence of one sensory input impacts on the development of other modalities (Gori, 2015). According to the cross-sensory calibration theory, in children younger than 8–10 years old, the most robust sensory modality calibrates the other ones (Gori et al., 2008). This suggests that specific sensory modalities can be more suitable than others to convey specific information and hence to teach specific concepts. For example, it was observed that children use the tactile modality to perceive the size of objects, whereas the visual signal is used to perceive their orientation (Gori et al., 2008). It was also observed that when the motor information is not available, visual perception of size is impaired (Gori et al., 2012) and that when visual information is not available, tactile perception of orientation of objects is impaired (Gori et al., 2010). These results suggest that until 8–10 years of age, sensory modalities interact and shape each other. Then, multisensory technology that exploits multiple senses can be crucial to communicating specific concepts in a more effective way (i.e., having multiple signals available, the child can use the one which is most suitable for the task). Scientific evidence also suggests that it is not always true that the lack of one sensory modality is associated with an enhancement of the remaining senses (Rauschecker and Harris, 1983; Rauschecker and Kniepert, 1994; Lessard et al., 1998; Röder et al., 1999, 2007; Voss et al., 2004; Lomber et al., 2010), but, in some cases, even the other not impaired senses are affected by the lack of the calibration modality (Gori et al., 2014; Finocchietti et al., 2015; Vercillo et al., 2015). For example, visually impaired children have impaired tactile perception of orientation (Gori et al., 2010); thus, touch cannot be used to communicate the orientation concept, and other signals, such as the auditory one, could be more suitable to convey this information.

We think that this scientific evidence should be reflected in teaching and learning practices, by introducing novel multisensory pedagogical methodologies grounded on it. In particular, we think that such scientific evidence supports an embodied and enactive pedagogical approach, using different sensory-motor signals and feedback (audio, haptic, and visual) to teach concepts to primary school children. For example, the use of sound associated with body movement could be an alternative way to teach visually impaired children the concept of orientation and angles. Such an approach would be more direct, i.e., natural and intuitive, since it is based on the experience and on the perceptual responses to motor acts. Moreover, the use of movement for learning was shown to deepen and strengthen learning, retention, and engagement (Klemmer et al., 2006; Habib et al., 2016).

It should be noticed that sensory combination and sensory integration are implemented differently in the way multisensory signals are provided through technology. At the technological level, there is a difference between teaching a concept by using more than one modality (i.e., by adopting multiple alternative strategies and promoting multisensory combination) versus stimulating those modalities simultaneously by providing redundant sensory signals (thus promoting multisensory integration). In the technological area, Nigay and Coutaz (1993) classified multimodal interactive systems depending on their use of modalities and on whether modalities are combined (i.e., what in computer science is called multimodal fusion). In particular, they made a distinction between sequential, simultaneous, and composite multimodal interactive systems. The kind of multimodal interactive system, which is selected to provide multisensory feedback, depends on and affects the pedagogical paradigm and the way the learning process develops. More research is needed to get a deeper understanding of all the implications related to this choice, e.g., with respect to the concepts to teach, the needs of teachers and students, the optimal way of providing technological support, the learning outcomes, and so on.

## Challenges

Multisensory technologies can help in overcoming the consolidated hegemony of vision in current educational practice. A too strong focus on one single sensory channel may compromise the effectiveness and personalization of the learning process. Moreover, a pedagogical approach based on one single modality may prevent the inclusion of children with impairments (e.g., with visual impairment).

More specifically, multisensory technologies can support the learning process by enhancing effectiveness, personalization, and inclusion. With respect to *effectiveness*, this may be affected by a wrong or an excessive usage of vision, which is not always the most suitable channel for communicating certain concepts to children.

As for *personalization*, a pedagogical methodology based almost exclusively on the visual modality would not consider the learning potential, and routes of access for learning in children, of exploiting the different modalities in ways that more comprehensively convey different kinds of information (e.g., the tactile modality is often better for perception of texture than vision). Moreover, we might speculate that a more flexible multisensory approach could highlight individual predispositions of children. It could be possible, for example, to observe a different individual tendency of preferring a specific learning approach for different children, demonstrating that specific sensory signals can be more useful for some children to learn specific concepts. For example, recent studies showed that musical training can be used as a therapeutic tool for treating children with dyslexia (e.g., Habib et al., 2016). The temporal and rhythmic features of music could indeed exert a positive effect on the multiple dimensions of the “temporal deficit” that is characteristic of some types of dyslexia. Other specific examples are autism and Attention Deficit Hyperactivity Disorder (ADHD). There is solid evidence that multisensory stimuli improve the accuracy of decisions. Can multisensory technology or stimuli also improve attention or learning speed or retention? In our opinion, this issue is worthy of further investigation and found evidence in this direction would be crucial for designing effective technological support for children with developmental disorders (ADHD, autism, specific learning disabilities, dyslexia, and so on).

Concerning *inclusion*, the lack of vision in children with visual disability impacts, e.g., the learning of geometrical concepts that are usually communicated through visual representations and metaphors. A delay in the acquisition of cognitive skills in visually impaired children directly affects their social competence, producing in turn feelings of frustration that represent a risk for the development of personality and emotional competence (Thompson, 1941). The use of multisensory technology would allow having the same method for teaching to be used by sighted and blind children, thus naturally breaking barriers among peers and facilitating social interactions.

## An Opportunity For Multisensory Technologies

In our view, multisensory technologies are ideal for effectively supporting a pedagogical approach exploiting the best-suited sensory modality to teach a concept.

**Figure 1:**
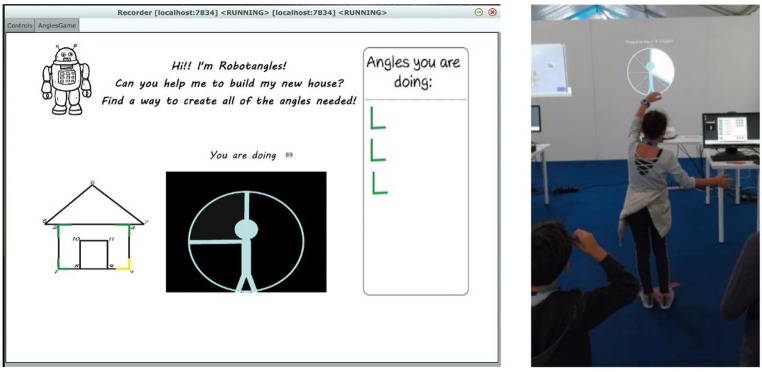
The visual feedback provided by a technology-mediated learning activity featuring angles. A child makes an angle by opening her arms, the arms representing the sides of the angle, and the head its vertex. Arms aperture is measured by a Microsoft Kinect v.2 device and mapped in real time onto visual and/or auditory feedback. The activity was designed and developed by the Casa Paganini – InfoMus research centre at DIBRIS – University of Genoa and the U-Vip Unit at Istituto Italiano di Tecnologia in the framework of the EU-H2020-ICT weDRAW project.

Multisensory technologies enable accurate and real-time mapping of motor behavior onto multiple facets of sound, music, tangible, and visual media, according to different strategies the teacher can select with great flexibility. Consider, for example, a recent technology-mediated learning activity we are developing for introducing geometric concepts, such as angles (Volta et al., 2018). In this activity, a child is asked to reproduce an angle by opening her arms. The child’s arms represent the two sides of the angle and her head its vertex. Arms aperture is automatically measured by means of a Microsoft Kinect v.2 device, and the motor behavior is mapped onto multisensory feedback in real time. A visual or auditory feedback or both of them is provided. The visual feedback consists of the visual representation within a circle of the angle the child is doing (see Figure 1). Concerning the auditory feedback, while the child moves her arms, she can listen to a musical scale covering the full range of angle amplitude. If the child changes the aperture of her arms, the note in the scale – played by a string instrument – changes according to the movement. A long distance between the arms (i.e., a big angle) corresponds to a low-pitch note, whereas a short distance (i.e., a small angle) corresponds to a high-pitch note. Such a mapping is grounded on psychophysical evidence showing that a low pitch is associated with a big size and a high pitch is associated with a small size (Tonelli et al., 2017). If the child is able to keep the same angle while rotating her arms, she listens to the same note with no changes in the auditory feedback, suggesting that angles are invariant under rotations. The teacher is provided with an interface enabling her to control the application (e.g., by selecting the angles that are proposed, the kind of feedback, several levels of difficulty, and so on). An initial and ongoing evaluation of this activity with children is suggesting that the proposed embodied representation of angles helps children in understanding angles and their properties (e.g., rotational invariance), even if more iterations of the development cycle are needed to address possible drawbacks (e.g., children get tired if asked to keep arms open for a too long time). While the angles activity implements a quite simple mapping of motor behavior onto visual and auditory feedback, more sophisticated approaches can be conceived. Multiple features of motor behavior can indeed be mapped onto multiple dimensions of sound morphology, including pitch, intensity, granularity, rhythm, and so on. While this is what usually happens when playing a musical instrument, technology makes it more flexible. Indeed, the teacher can choose which motor features are mapped onto which sound parameters and the child can quickly achieve a fine-grained control on the sound parameters, something that would require many years of practice with a traditional musical instrument. These issues have been debated for a long time in the literature of sound and music computing, see for instance, Hunt and Wanderley (2002), for a seminal work on this topic and the series of conferences on New Interfaces for Musical Expression^2^.

In our view, the adoption of an embodied and enactive pedagogical approach, tightly integrated with multisensory technology, would, therefore, foster effectiveness (for each specific concept, the most suited modality can be exploited) and personalization (flexibility for teachers and students) in the learning process. Moreover, inclusion can also take a great advantage: teaching can exploit the most suited substitutive modality for impaired children.

## Guidelines

Since big opportunities most often entail likewise big risks, the introduction of multisensory technologies in the classroom needs to be careful. From our experience in technology-enhanced learning projects, we propose six golden rules we deem important for catching this opportunity and fully exploiting it.

**Figure 2:**
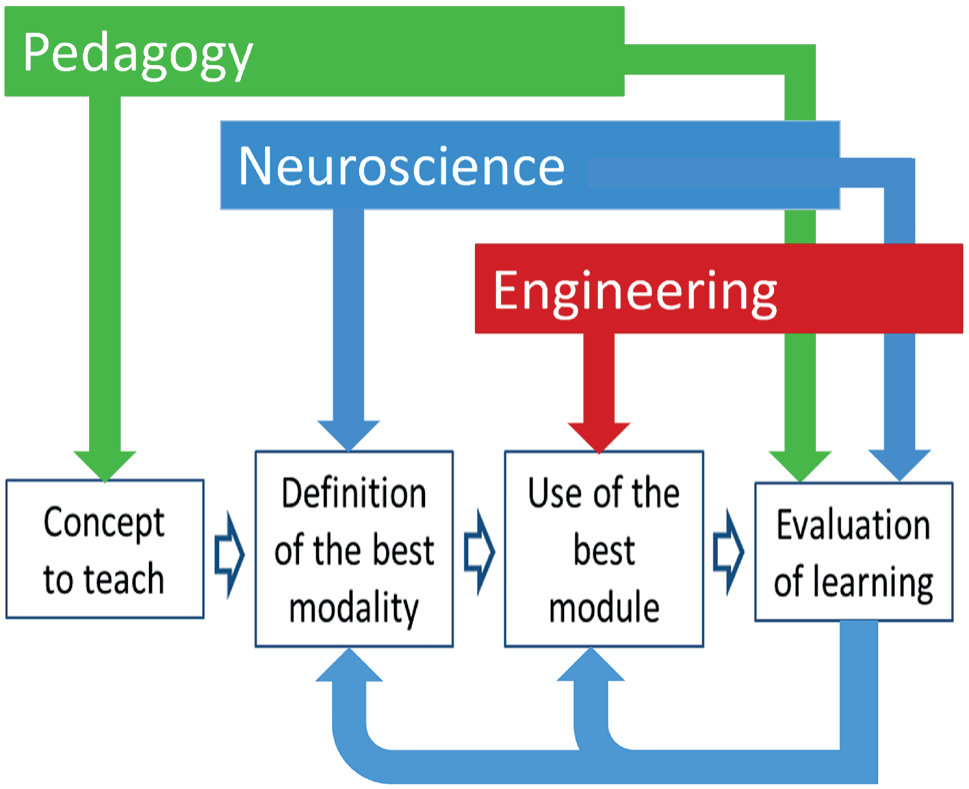
A four-phase iterative methodology directly involving the teacher in the choice of the sensory modalities to be exploited for conveying a specific concept. Following an initial evaluation phase, the best modality to teach the concept is identified for each child in a personalized way, and the selected modality is used to teach further concepts. The methodology involves tight integration of pedagogical, neuroscientific, and technological knowledge and an effective multidisciplinary approach.

1. *Ground technology on pedagogical needs*. Multisensory technologies should be tailored to the pedagogical needs of teachers. That is, they can help with teaching concepts that teachers specifically deem relevant in this respect. These could be concepts that are particularly difficult to understand for children or concepts that may enjoy communication through a sensory modality other than vision. We recently conducted a survey on over 200 math teachers. It was surprising for us to see that more than 75% of teachers agreed on the same concepts as the most difficult for children and the most appropriate for technological intervention.
2. *Ground technology on scientific evidence*. Multisensory technology should leverage on sensorial, perceptual, and cognitive capabilities children have according to scientific evidence. Concretely, for example, a technology able to detect specific motor behaviors in a target population of children (e.g., primary school) makes sense only if scientific evidence shows that children in the target population can actually display such behaviors. The same holds for feedback: multisensory technology can provide a specific feedback (e.g., based on pitch), if (1) children can perceive it (e.g., they developed perception of pitch) and (2) an experimentally proven association exists between feedback and concept to be communicated (e.g., the association between pitch and size of objects).
3. *Adopt an iterative design approach and rigorously assess learning outcomes*. Iterative design and refinement of technology are a critical component in the development cycle of interactive technologies. In case of multisensory technologies for education, this is crucial for successfully integrating technology in the classroom. Each iteration in the development process needs to rigorously assess learning outcomes (e.g., the speed of learning, longer-term outcomes like knowledge retention, student-centered outcomes like learner satisfaction, classroom behavior, and so on) and use this feedback to inform the next iteration.
4. *Make technology flexible and customizable.* This is a typical goal for technologies, but it assumes here a particular relevance. It means assessing (1) which is the preferred sensory modality for a child to learn a specific concept and (2) whether specific impairments require exploiting particular sensory modalities. As a side effect, technology may help with screening for behavioral problems and addressing them. For example, recent studies show that musical training can be used as a therapeutic tool for treating children with dyslexia (e.g., Curzon et al., 2009).
5. *Emphasize the role of the teacher*. In our view, technology does not replace the teacher. Rather, the teacher plays the central role of *mediator*. In an iterative methodology (see Figure 2), the teacher first chooses a concept to teach; then, following an initial evaluation phase, she identifies the best modality to teach it to each child and personalizes technology to exploit the selected modality; she finally evaluates the outcomes of the learning process and adopts possible further actions. Moreover, design, development, and evaluation of technology should be obviously carried out in the framework of a participatory design process involving teachers and students (see Guideline 3).
6. *Promote cross-fertilization with the arts and human sciences*. Taking a rigorous scientific approach should not exclude the opportunity of getting inspiration from humanities, and in particular from arts. Recent initiatives (e.g., the EU STARTS platform^3^) witness the increased awareness of how art and science are two strongly coupled aspects of human creativity (Camurri and Volpe, 2016), as well as the impact of art on scientific and technological research. In case of multisensory technology for education, the extraordinary ability art has of conveying content by means of sound, music, and visual media provides, in our view, a significant added value.

## Conclusion

We developed and tested our approach in the framework of the weDRAW project^4^. This was an EU-H2020-ICT-funded project focusing on multisensory technologies for teaching math to primary school children. The final goal was to open a new teaching/learning channel based on multisensory interactive technology. The project represented an ideal testbed to assess the support of multisensory technology to learning math. More importantly, we think that the approach we outlined in this article can enable the development of a multisensory embodied and enactive learning paradigm and of a teaching ecosystem that applies in the same way and provides the same opportunities to both typically developed and impaired children, thus breaking the barriers between them and fostering inclusion.

## Ethics Statement

This is a perspective paper, presenting our viewpoint on future research that will potentially involve human subjects. The perspective paper, however, is not based on any specific experimental study but is rather grounded on the existing literature and on our vision of future research.

## Author Contributions

GV and MG equally contributed to both the concept of the article and its writing.

### Conflict of Interest Statement

The authors declare that the research was conducted in the absence of any commercial or financial relationships that could be construed as a potential conflict of interest.

## Footnotes

^1^See e.g., http://www.pcadvisor.co.uk/feature/software/best-maths-apps-for-children-3380559/

^2^http://www.nime.org

^3^https://ec.europa.eu/digital-single-market/en/ict-art-starts-platform

^4^http://www.wedraw.eu
